# One-year multicenter prospective real-world study of vericiguat effectiveness and safety in Spain

**DOI:** 10.1093/eschf/xvag138

**Published:** 2026-05-13

**Authors:** Alberto Esteban-Fernández, Beatriz Calvo-Bernal, Raquel López-Vilella, Gregorio de Lara, Laia C Belarte-Tornero, Moisés Barrantes-Castillo, Inés Gómez-Otero, Julio Nuñez-Villota, Carolina Robles-Gamboa, José López-Aguilera, Ana Torremocha-López, María Melendo-Viu, Clara Simón-Ramón, Paula Fluvià-Burgués, Francisco J Pastor-Pérez, Gonzalo Alonso-Salinas, José Manuel García-Pinilla, Antonia Pomares-Varó, Jara Gayán-Ordás, David González-Calle, Pablo Díez-Villanueva, Mario Galván-Ruiz, Carolina Ortiz-Cortés, Pedro Caravaca-Pérez, Cristina Goena-Vives, Julia Seller, Marta Jiménez-Blanco, Pau Codina, José María Vietiez, Laura Jordán-Martínez, Maria Thiscal López-Lluva, Elisabet Mena-Sebastià, Pedro Pájaro, Alejandro Recio-Mayoral, VERISEC investigators, Francisco Camacho Jurado, Ángel Iniesta Manjavacas, Sonia Mirabet, Silvia López Fernández, Juan Luis Bonilla Palomas, Marta Cobo-Marcos, Sara Corredera García, Vanesa Escolar, Juan Górriz-Magaña, Ainara Lozano, Aleix Olivella, Esther Sánchez Corral, Mikel Taibo-Urquía, Francisco Bermúdez-Jiménez, Ricardo Martínez-Fernández

**Affiliations:** Cardiology Department, Hospital Universitario Severo Ochoa, Calle Orellana s/n, Leganés, Madrid 28911, Spain; Facultad de Ciencias Biomédicas y de la Salud, Universidad Alfonso X El Sabio, Avenida Universidad, 1, 28691 Villanueva de la Cañada, Madrid, Spain; Cardiology Department, Hospital Puerto Real, Cádiz, Spain; Cardiology Department, Hospital Universitari La Fe, Valencia, Spain; Cardiology Department, Hospital de Torrevieja, Alicante, Spain; Cardiology Department, Hospital del Mar, Barcelona, Spain; Heart Diseases Biomedical Research Group (GREC), IMIM (Hospital del Mar Medical Research Institute), Barcelona, Spain; Department of Medicine, School of Medicine, Universidad Autonoma de Barcelona, Barcelona, Spain; Cardiology Department, Hospital de Palamós, Girona, Spain; Cardiology Department, Hospital Clínico de Santiago de Compostela, A Coruña, Spain; Centro de Investigación Biomédica en Red Cardiovascular (CIBERCV), Madrid, Spain; Cardiology Department, Hospital Clínico de Valencia, Valencia, Spain; Institute of Health Research-INCLIVA, Valencia, Spain; Cardiology Department, Hospital de Toledo, Toledo, Spain; Cardiology Department, Hospital Reina Sofía, Córdoba, Spain; Cardiology Department, Hospital Universitario La Paz, Madrid, Spain; Cardiology Department, Hospital Universitario Álvaro Cunqueiro, Vigo, Spain; Cardiology Department, Hospital Sant Pau, Barcelona, Spain; Institut de Recerca Sant Pau, Barcelona, Spain; Cardiology Department, Hospital Dr Trueta, Girona, Spain; Cardiology Department, Hospital Virgen de la Arrixaca, Murcia, Spain; Cardiology Department, Hospital Universitario de Navarra, Pamplona, Spain; Navarrabiomed, Instituto de Investigación en Salud (IdISNa), Pamplona, Spain; Centro de Investigación Biomédica en Red Cardiovascular (CIBERCV), Madrid, Spain; Cardiology Department, Hospital Universitario Virgen de la Victoria, Málaga, Spain; IBIMA-Plataforma BIONAND, Málaga, Spain; Departamento de Medicina y Dermatología, Universidad de Málaga, Málaga, Spain; Cardiology Department, Hospital Universitari Mutua Terrassa, Barcelona, Spain; Cardiology Department, Hospital Arnau de Vilanova de Lleida, Lleida, Spain; Institut de Recerca Biomèdica de Lleida, Lleida, Spain; Cardiology Department, Hospital Universitario de Salamanca, Salamanca, Spain; Cardiology Department, Hospital La Princesa, Madrid, Spain; Cardiology Department, Hospital Dr Negrin, Las Palmas, Spain; Cardiology Department, Hospital Fundación de Alcorcón, Madrid, Spain; Cardiology Department, Hospital Clinic de Barcelona, Barcelona, Spain; Cardiology Department, Hospital Donostia, San Sebastián, Spain; Cardiology Department, Hospital de Denia, Alicante, Spain; Centro de Investigación Biomédica en Red Cardiovascular (CIBERCV), Madrid, Spain; Cardiology Department, Hospital Universitario Ramón y Cajal, Madrid, Spain; Cardiology Department, Hospital Germans Trias I Pujol, Barcelona, Spain; Cardiology Department, Hospital Lucus Augusti, Lugo, Lugo, Spain; Cardiology Department, Hospital Virgen de las Nieves, Granada, Spain; Cardiology Department, Hospital Universitario de León, León, Spain; Cardiology Department, Hospital Moisés Broggi, Barcelona, Spain; Cardiology Department, Hospital Juan Ramón Jiménez, Huelva, Spain; Cardiology Department, Hospital Virgen Macarena, Sevilla, Spain

**Keywords:** Heart failure, Hospitalization, Natriuretic peptides, Vericiguat, Worsening heart failure

## Abstract

**Aims:**

To evaluate the effectiveness and safety of vericiguat in patients with heart failure and reduced ejection fraction (HFrEF) following recent decompensation in routine clinical practice in Spain.

**Methods:**

VERISEC is a prospective, multicentre registry of 835 consecutive patients initiating vericiguat at 41 centres in Spain. Functional class, biochemical markers, ventricular function, and clinical events were analysed during a 1-year follow-up.

**Results:**

Patients (age 71.3 years [SD: 11.2], 78.9% male) received highly optimized baseline therapy: 91.5% SGLT2i, 90.7% beta-blockers, 85.4% RAASi, and 79.8% MRAs. Quadruple therapy remained stable (61.7% baseline to 63.0% at 12 months; *P* = .526). At 1-year follow-up, significant improvements were observed. NT-proBNP decreased from 3532.0 (IQR: 1689.3–6974.5) to 2291.5 pg/ml (IQR: 1063.0–5076.3; *P* < .001) and LVEF increased from 30.3% (SD: 7.6) to 35.4% (SD: 11.1; *P* < .001). NYHA class improved, with class II patients increasing from 55.6% to 62.2% (*P* < .001). Mean HF decompensations requiring intravenous diuretics decreased from 1.34 (SD: 1.1) in the preceding year to 0.65 (SD: 1.3) during follow-up. Non-HF cardiovascular hospitalizations decreased from 0.98 (SD: 1.4) to 0.39 (SD: 1.0). Vericiguat was discontinued in 13.4% of patients, primarily due to symptomatic hypotension (49.4%). Higher baseline NT-proBNP (per 1000-pg/ml increase) independently predicted discontinuation (OR: 1.06; 95% CI: 1.03–1.08; *P* < .001).

**Conclusions:**

Vericiguat added to optimized quadruple therapy was associated with reverse remodelling, reduced NT-proBNP, improved functional class, and a numerical reduction in HF-related events in real-world HFrEF patients. These findings confirm the clinical utility and safety of vericiguat in routine practice.

## Graphical Abstract

**Graphical Abstract:**
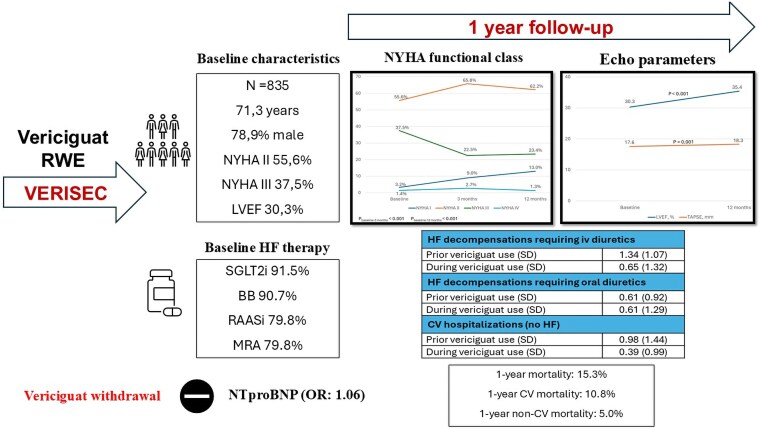

## Introduction

Heart failure (HF) remains a major cause of morbidity and mortality worldwide. Although 1-year mortality has declined to around 30% due to improvements in diagnosis and treatment, the burden of disease remains substantial, reflected by frequent hospitalizations and high healthcare costs that adversely affect patients’ quality of life.^1–3^

Worsening HF has traditionally required hospitalization owing to more severe signs and symptoms of HF or the need for urgent intravenous diuretics. More recently, some researchers have suggested expanding this definition to include individuals who require urgent increases in oral diuretics.^4^ These worsening episodes, which often interrupt apparently stable periods, are associated with a higher likelihood of mortality and reduced functional capacity, highlighting the need for prompt management to mitigate adverse outcomes.^5,6^

Current guideline-directed medical therapy (GDMT) for patients with reduced ejection fraction (HFrEF) includes the four foundational pillars: renin–angiotensin–aldosterone system inhibitors (RAASi) (preferably angiotensin receptor–neprilysin inhibitors), beta-blockers (BB), mineralocorticoid receptor antagonists (MRA), and sodium-glucose co-transporter 2 inhibitors (SGLT2i).^7,8^ Despite their proven benefits, implementation remains suboptimal in routine clinical practice.^9,10^ Moreover, even with comprehensive quadruple therapy, many of these patients continue to face high risks of disease progression.^11^ This residual risk underscores the need for new therapeutic strategies, such as the soluble guanylate cyclase stimulator vericiguat.

The VICTORIA trial demonstrated that vericiguat, when added to GDMT in patients with a recent WHF event, reduced the risk of cardiovascular death or HF hospitalization.^12^ Furthermore, a recent meta-analysis suggested that adding vericiguat to standard HF treatment vs. placebo may reduce all-cause mortality by 59%, cardiovascular mortality by 65%, and combined cardiovascular death or admission for HF by 57%.^13^ While clinical trial data are valuable, real-world evidence is useful to confirm these findings in daily practice, as most available evidence is currently based on retrospective studies or small samples.^14–25^ This study (VERISEC) aimed to describe the clinical profile of patients initiating vericiguat, assess GDMT optimization, and evaluate safety and clinical outcomes during a 12-month follow-up.

## Methods

### Study population

VERISEC was a multicentre, national, prospective observational registry study. All patients initiating treatment with vericiguat according to guideline recommendations and the attending physician’s judgment in clinical practice at 41 centres in Spain were consecutively enrolled between December 2022 (the date of commercialization in Spain) and October 2023. To ensure a well-defined cohort of worsening HF, patients had to fulfil specific inclusion criteria: symptomatic HFrEF with a recent decompensation requiring either hospitalization within the previous 6 months or intravenous diuretic therapy in an outpatient setting within the previous 3 months, along with elevated natriuretic peptides. The baseline methodology and clinical characteristics of VERISEC have been published elsewhere.^11^ Vericiguat was prescribed in addition to other standard HF treatments, including RAASi, BB, MRA, and SGLT2i, following stabilization of volume status and optimization of diuretic therapy and in accordance with the summary of product characteristics and guidelines, as well as the attending physicians’ judgment. Patients were monitored for 12 months after initiating vericiguat. The study was approved by the Research Ethics Committee of Severo Ochoa University Hospital and endorsed by the ethics committees of all participating centres.

Study data were sourced from medical records and stored digitally at each centre in accordance with local procedures. The clinical and additional variables assessed at baseline were as follows: biodemographic characteristics and medical history; findings from physical examination; details of HF history; New York Heart Association (NYHA) functional class; echocardiographic measurements; HF treatments, with dosages reported as a percentage of maximum recommended doses based on current guidelines; use of cardiac resynchronization therapy and defibrillators; hospitalizations or emergency department visits within the previous 12 months; and laboratory parameters including renal function, haemoglobin, and electrolytes.

HF treatments, target doses, NYHA class, and physical, laboratory, and echocardiographic assessments were evaluated at 3 and 12 months of therapy. Target doses for GDMT were explicitly defined according to the 2021 HF European Guidelines^7^ (e.g. 50 mg daily for MRAs, 10 mg daily for bisoprolol, and 97/103 mg twice daily for sacubitril/valsartan). Additionally, clinical events were documented at 3 and 12 months. These included the frequency of HF decompensations requiring intravenous diuretics or an increase in oral diuretics, the number of hospital admissions for cardiovascular (non-HF) and non-cardiovascular causes, and incidences of cardiovascular and non-cardiovascular mortality.

Adverse effects associated with vericiguat were assessed at 3 and 12 months, focusing on symptomatic hypotension, syncope, patient decision, digestive disorders, and impaired kidney function.

### Statistical analysis

The normality of the data distribution was assessed using the Kolmogorov–Smirnov test. Quantitative variables were reported as means and standard deviations or medians and interquartile ranges (IQR) as appropriate, while categorical data were summarized as frequencies and percentages. The type of variable and its distribution determined the selection of statistical tests: hypothesis testing for paired samples was employed to evaluate changes across visits (baseline to 3 months and baseline to 12 months). For continuous variables, either the Student's *t* test or the Wilcoxon signed-rank test was used. For categorical variables, the chi-square test or Fisher's exact test was implemented as appropriate. Event rates before and after the initiation of vericiguat therapy were compared over a 12-month period. A binary logistic regression model was developed to analyse the predictors of drug discontinuation. All clinically relevant variables and those with *P* < .10 in the univariate analysis were included in the maximum model. The backward elimination method was used to select the final model. Data were processed and analysed using STATA, version 17.0.

## Results

A total of 835 patients were included. The mean age was 71.3 (SD: 11.2) years, and 78.9% were male. At baseline, systolic blood pressure was 116.2 (SD: 17.6) mmHg, estimated glomerular filtration rate (eGFR) was 49.0 (IQR: 34;67) ml/min, and N-terminal pro-brain natriuretic peptide (NT-proBNP) was 3532.0 (IQR: 1689.3;6974.5) pg/ml. Mean left ventricular ejection fraction was 30.3% (7.6%), and severe mitral regurgitation was observed in 27.4%. During the month before inclusion, 55.2% had experienced HF decompensation; 24.3% did so within the preceding 3 months (*Table 1*).

**Table 1:** Baseline clinical characteristics

Characteristics	VERISEC *n* = 835
Clinical characteristics	
Age, years (SD)	71.3 (11.2)
Sex (male), (%)	659 (78.9)
Hypertension, *n* (%)	627 (75.1)
Dyslipidaemia, *n* (%)	572 (68.5)
Diabetes, *n* (%)	431 (51.6)
Smoking, *n* (%)	
Never	310 (37.1)
Former	425 (50.9)
Current	98 (11.7)
Chronic obstructive pulmonary disease, *n* (%)	132 (15.8)
Asthma, *n* (%)	13 (1.6)
Clinical parameters	
Systolic blood pressure, mmHg (SD)	116.2 (17.6)
Diastolic blood pressure, mmHg (SD)	68.8 (12.0)
Heart rate, bpm (SD)	71.0 (12.4)
**HF history**	
HF aetiology, *n* (%)	
Ischemic	419 (50.2)
Dilated cardiomyopathy	227 (27.2)
Valvular	57 (6.8)
Toxicity	36 (4.3)
Familial	28 (3.4)
Tachycardia-induced cardiomyopathy	23 (2.8)
Other	40 (4.8)
Inclusion setting, *n* (%)	
Outpatient	721 (86.3)
Emergency department	2 (0.2)
Hospitalization	112 (13.4)
Index episode, *n* (%)	
HF decompensation in the previous 1 month	472 (56.5)
HF decompensation in the previous 3 months	203 (24.3)
HF decompensation in the previous 6 months	160 (19.2)
Functional class^a^, *n* (%)	
NYHA I	25 (3.2)
NYHA II	447 (55.6)
NYHA III	291 (37.5)
NYHA IV	11 (1.4)
Echocardiogram parameters	
LVEF, % (SD)	30.3 (7.6)
TAPSE, mm (SD)	17.6 (4.0)
Severe MR (III or IV), *n* (%)	229 (27.4)
Severe TR (III or IV), *n* (%)	139 (16.6)
Moderate to severe pulmonary hypertension^b^, *n* (%)	219 (26.2)
**HF therapy**	
SGLT2i, *n* (%)	764 (91.5)
Beta blockers, *n* (%)	757 (90.7)
Renin–angiotensin–aldosterone system inhibitors, *n* (%)	713 (85.4)
MRA, n (%)	666 (79.8)
Loop diuretics, *n* (%)	733 (87.8)
Current intermittent outpatient levosimendan, *n* (%)	132 (15.8)
ICD or CRT^c^, *n* (%)	374 (44.8)
Initial dose of vericiguat, *n* (%)	
2.5 mg	799 (95.7)
5 mg	33 (4.0)
10 mg	3 (0.4)

COPD, chronic obstructive pulmonary disease; CRT, cardiac resynchronization therapy; CV, cardiovascular; HF, heart failure; ICD, implantable cardioverter defibrillator; LVEF, left ventricular ejection fraction; MR, mitral regurgitation; NYHA, New York Heart Association; SD, standard deviation; SGLT2i, sodium-glucose co-transporter 2 inhibitors; TAPSE, tricuspid annular plane systolic excursion; TR, tricuspid regurgitation.

^a^Data available for 776 patients.

^b^Moderate-to-severe pulmonary hypertension was defined as a systolic pulmonary artery pressure >45 mmHg, as estimated by the tricuspid regurgitation peak velocity and the assessment of the inferior vena cava diameter and collapsibility.

^c^Including CRT with a pacemaker or defibrillator.

Regarding HF therapy at baseline, 91.5% of patients were receiving SGLT2i, 90.7% BB, 85.4% RAASi, and 79.8% MRA. Vericiguat was started in 86.3% of patients in the outpatient clinic after decompensation and in 13.4% during hospitalization. (*Table 1*). The proportion of patients taking SGLT2i, BB, RAASi, and MRA generally remained stable during the follow-up (*Table 2*).

**Table 2:** Heart failure treatments at one-year follow-up with vericiguat

	Baseline	3 months^a^	*P*_baseline-3 months_	12 months^b^	*P*_baseline-12 months_
RAASi, *n* (%)	713 (85.4)	574 (86.6)	.871	506 (86.6)	1.000
BB, *n* (%)	757 (90.7)	612 (92.3)	.851	533 (91.3)	1.000
MRA, *n* (%)	666 (79.8)	544 (82.1)	.066	463 (79.3)	.911
SGLT2i, *n* (%)	764 (91.5)	609 (91.9)	.690	518 (88.7)	.049
Quadruple therapy	515 (61.7)	432 (65.1)	.119	368 (63.0)	.526
Loop diuretics, *n* (%)	733 (87.8)	573 (86.4)	.306	490 (83.9)	.230
Loop diuretic dose, mg (SD)	68.2 (41.8)	68.9 (45.6)	.530	68.2 (44.9)	.370

Quadruple therapy comprised RAASi + BB + MRA + SGLT2i. RAASi, renin-angiotensin-aldosterone system inhibitors; BB, beta blockers; MRA, mineralocorticoid receptor antagonists; SGLT2i, sodium-glucose co-transporter 2 inhibitors.

^a^Data available for 663 patients.

^b^Data available for 584 patients.

**Figure 1:**
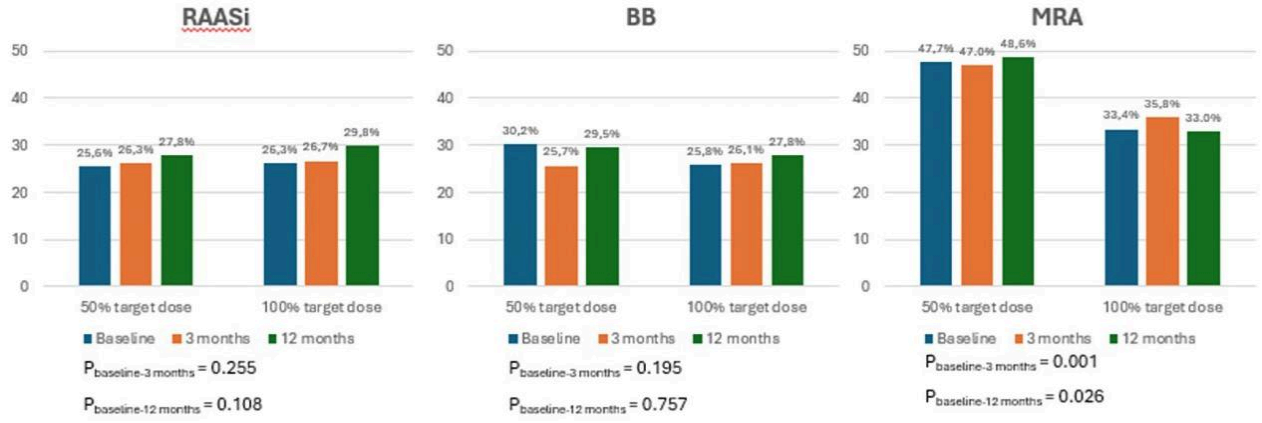
Target doses of RAASi, BB, and MRA were achieved over 1 year of treatment with vericiguat. RAASi, renin–angiotensin–aldosterone system inhibitors; BB, beta blockers; MRA, mineralocorticoid receptor antagonists

The proportion of patients taking SGLT2i, BB, RAASi, and MRA remained stable during follow-up, with no significant changes observed in any individual drug class (*Table 2*). Consistent with this, the concurrent use of quadruple therapy remained stable throughout the study period, with 61.7% of patients receiving the four-drug combination at baseline and 63.0% at 12 months (*P* = .526). Regarding dosing, no significant changes were observed in the target doses of BB and RAASi, and the distribution of MRA dosing remained consistent (*Figure 1*), reflecting the maintenance of high-quality baseline optimization during the study period.

By study end, NYHA functional class had improved significantly over baseline (*P* < .001), with most patients shifting towards lower classes; changes were already evident at month 3 (Figure S1). A modest but significant reduction in systolic blood pressure was observed during follow-up, while heart rate remained stable. Changes in laboratory parameters such as haemoglobin and electrolytes were not relevant, whereas both eGFR and NT-proBNP decreased significantly over time (*Table 3*).

**Table 3:** Clinical and laboratory parameters after 1 year of treatment with vericiguat

	Baseline	3 months	*P*_baseline-3 months_	12 months	*P*_baseline-12 months_
SBP, mmHg (SD)	116.2 (17.6)	111.9 (17.3)	<.001	114.1 (17.8)	<.001
DBP, mmHg (SD)	68.8 (12.0)	65.9 (11.9)	<.001	67.1 (12.2)	<.001
HR, bpm (SD)	71.0 (12.4)	69.3 (12.3)	.002	69.7 (11.8)	.060
Haemoglobin, g/dL (IQR)	13.8 (12.3;15.1)	13.6 (12.0;15.0)	.023	13.9 (12.2;15.3)	.416
Ferritin, ng/mL (IQR)	185 (99;360)			201 (98;340)	.648
Transferin saturation, % (IQR)	22 (16;29)			24 (18;39)	.001
Creatinine, mg/dl (IQR)	1.4 (1.0;1.8)	1.4 (1.0;2.0)	<.001	1.4 (1.1;1.9)	<.001
eGFR, mL/min (IQR)	49 (25;67)	46 (32;64)	<.001	45 (32;65)	<.001
Sodium, mmol/l (IQR)	140 (138;142)	140.0 (138;142)	<.001	141 (138;143)	.157
Potassium, mmol/l (IQR)	4.4 (4.1;4.8)	4.4 (4.1; 4.8)	.**150**	4.4 (4.1;4.8)	.160
NT-proBNP, pg/ml (IQR)	3532.0 (1689.3;6974.5)	2988.0 (1338.5;5871.5)	.018	2291.5 (1063.0;5076.3)	<.001

DBP, diastolic blood pressure; eGFR, estimated glomerular filtration rate; HR, heart rate; IQR, interquartile range; SBP, systolic blood pressure; SD, standard deviation; NT-proBNP, N-terminal pro-brain natriuretic peptide.

Echocardiographic assessment revealed significant improvements at 12 months. Left ventricular ejection fraction (LVEF) increased from a mean of 30.3% (SD: 7.6) at baseline to 35.4% (SD: 11.1) at 12 months (*P* < .001). Tricuspid annular plane systolic excursion (TAPSE) improved from a mean of 17.6 mm (SD: 4.0) at baseline to 18.3 mm (SD: 3.9) at 12 months (*P* < .001), demonstrating favourable reverse remodelling. No relevant changes were observed in other echocardiographic parameters (Figure S2).

**Figure 2:**
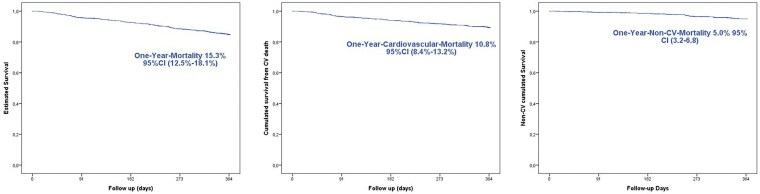
Survival analysis over 1 year of treatment with vericiguat. CV, cardiovascular

During the first year of follow-up of treatment with vericiguat, 105 patients (12.6%) died. Of these, 74 deaths (8.9%; 70.5% of total deaths) were attributed to cardiovascular causes (*Figure 2*). During the 12-month follow-up, the mean number of HF decompensations requiring intravenous diuretics was 0.65 (SD: 1.32), whereas the mean number in the year prior to enrolment was 1.34 (SD: 1.07). Similarly, the mean number of non-HF cardiovascular hospitalizations was 0.39 (SD: 0.99) during follow-up (compared to 0.98 [SD: 1.44] in the previous year), and non-cardiovascular hospitalizations were 0.29 (SD: 0.63) (compared to 0.47 [SD: 1.01] in the preceding year) (*Table 4*).

**Table 4:** Clinical events in the year prior to enrolment and during 12-month follow-up

Event	Year prior to enrolment	12-month follow-up
HF decompensations (intravenous diuretics)	1.34 (1.07)	0.65 (1.32)
HF decompensations (oral diuretics)	0.61 (0.92)	0.61 (1.29)
CV hospitalizations (non-HF)	0.98 (1.44)	0.39 (0.99)
Non-CV hospitalizations	0.47 (1.01)	0.29 (0.63)

Data are presented as mean (standard deviation). CV, cardiovascular; HF, heart failure. Comparison of event rates is descriptive; no formal statistical inference is claimed due to the observational nature of the study and the inclusion criteria requiring a recent worsening HF event.

Most patients (95.7%) initiated vericiguat at a dose of 2.5 mg. Treatment was discontinued in 8.8% of patients by month 3 and in 13.4% by month 12. Among those who continued therapy, 55.1% and 64.1% reached the 10-mg target dose at 3 and 12 months, respectively (*P* < .001). Symptomatic hypotension was the leading cause of discontinuation (52.2% at 3 months; 49.4% at 12 months), followed by impaired kidney function (23.9% and 23.5%, respectively) (*Table 5*).

**Table 5:** Treatment with vericiguat after 1 year of follow-up

	Baseline	3 months^a^	*P*_baseline-3 months_	12 months^b^	*P*_baseline-12 months_
Dose of vericiguat, *n* (%)					
2.5 mg	799 (95.7)	111 (14.6)	<.001	59 (9.3)	
5 mg	33 (4.0)	164 (21.5)		84 (13.2)	<.001
10 mg	3 (0.4)	420 (55.1)		407 (64.1)	
Withdrawal, *n* (%)	0	67 (8.8)	—	85 (13.4)	—
Reason for withdrawal^c^, *n* (%)					
Symptomatic hypotension		35 (52.2)		42 (49.4)	
Syncope		1 (1.5)		4 (4.7)	
Patient decision		12 (17.9)		7 (8.2)	
Digestive disorder		7 (10.4)		4 (4.7)	
Impaired kidney function		16 (23.9)		20 (23.5)	
Other	—	8 (11.9)	—	8 (9.4)	—

^a^Data available for 762 patients.

^b^Data available for 635 patients (within 1 year of follow-up, 108 patients had died, and 67 patients had discontinued vericiguat by the third month).

^c^One patient could have more than one reason for withdrawal.

In the regression model, NT-proBNP was identified as a predictor of vericiguat withdrawal over 1 year of follow-up [OR 1.06; 95% CI 1.03–1.08], with each 1000-pg/ml increase in NT-proBNP associated with a 6% higher probability of discontinuation. Conversely, age [OR 0.98; 95% CI 0.96–0.99], systolic blood pressure [OR 0.98; 95% CI 0.97–0.99], and heart rate [OR 0.98; 95% CI 0.96–0.99] were protective factors, each associated with a 2% lower probability of vericiguat discontinuation per unit increase. Thus, older patients and those with higher systolic blood pressure or heart rate were less likely to discontinue vericiguat.

## Discussion

VERISEC is one of the largest prospective real-world studies of vericiguat to date. In this cohort of patients with HFrEF and recent decompensation, vericiguat initiation—added to a stable and highly optimized background therapy—was associated with significant improvements in cardiac function and NT-proBNP levels. While we observed a favourable clinical course with a low numerical incidence of HF events, these findings should be interpreted with caution. Nonetheless, our results underscore the clinical relevance of prospective studies in evaluating vericiguat in routine practice, highlighting its safety and applicability as part of comprehensive management for high-risk patients already receiving GDMT.^14–26^

Our patient cohort had more comorbidities and received more GDMT than those in the VICTORIA trial.^11,12^ Real-world studies show significant variation in patient characteristics, such as age, systolic blood pressure, LVEF, NT-proBNP levels, and NYHA class, among those treated with vericiguat.^14–25^ Overall, our findings differ clinically from the VICTORIA trial, as do those from real-world studies, likely owing to small sample sizes.

Approximately 92% of patients received SGLT2i, 91% BB, 85% RAASi, and 80% MRA, compared with 2.7%, 93%, 88%, and 70%, respectively, in the VICTORIA trial.^14^ Real-world studies suggest that GDMT is more frequent than in VICTORIA and aligns with our observations,^14–25^ reflecting a rising trend in implementation of therapy. This distinction is crucial because, unlike the VICTORIA trial, where SGLT2i and ARNI use was marginal, our cohort reflects contemporary practice. The improvements in NT-proBNP and LVEF despite nearly universal quadruple therapy suggest a synergistic effect of the sGC-cGMP pathway that extends beyond current standard-of-care. This scenario was not fully captured in the pivotal trial. Nonetheless, a substantial proportion of HF patients remain at risk for complications.^9,10^ Some studies indicate that vericiguat may facilitate the optimization of other HF medications through complementary mechanisms, contributing to improvement in symptoms.^15,17,24^

Consistent with the high-quality care in specialized units, our cohort demonstrated exceptionally high baseline optimization of GDMT, which remained stable throughout the study. Unlike other registries where follow-up involves significant drug titration, the concurrent use of quadruple therapy in VERISEC remained consistent from baseline to 12 months. This stability is particularly relevant, as it suggests that the observed improvements in LVEF and NT-proBNP are likely associated with the initiation of vericiguat rather than incremental optimization of background therapy. These findings underscore that vericiguat provides additional clinical benefit even when added to a highly optimized therapeutic framework in patients following a recent decompensation.

In the VERISEC study, patients showed improvements in functional class. Although the VICTORIA trial reported no notable differences in Kansas City Cardiomyopathy Questionnaire scores compared with placebo, the benefits of vericiguat were evident across different baseline health statuses.^12,27^ Observational studies further indicate potential improvements in functional class and quality of life with vericiguat.^14,15,21,23^ GDMT reduces NT-proBNP levels, correlating with better outcomes, and our study also observed a steady decline in NT-proBNP.^28^ Most real-world evidence indicates that vericiguat lowers NT-proBNP levels, although variability is likely due to differences in baseline levels.^14,16,19–21^ Notably, efficacy was reduced for patients with very high NT-proBNP in the VICTORIA trial,^29,30^ and in our cohort, higher baseline NT-proBNP was associated with an increased likelihood of discontinuing vericiguat, suggesting that very elevated peptide levels may limit treatment persistence.

In patients with HFrEF, vericiguat has been associated with reverse remodelling and improvements in both left and right ventricular ejection fractions.^31^ These observations are consistent with the VICTORIA echocardiographic sub-study and real-world data, irrespective of concurrent HF therapy and renal function,^20,21,23,31–33^ suggesting favourable effects on the pathophysiology of HF. In chronic HF, such echocardiographic improvements, particularly in ventricular function, may underlie benefits, including better functional class, reduced hospitalizations, and potentially improved survival.

In this study, 13% of patients died, with 71% of deaths attributed to cardiovascular causes. By comparison, in the VICTORIA trial (median follow-up 10.8 months), mortality was 20.3% for vericiguat and 21.2% for placebo, with most deaths also due to cardiovascular causes.^12^ In our cohort, the incidence of HF decompensations requiring intravenous diuretics and cardiovascular hospitalizations during follow-up was numerically lower than in the year prior to inclusion. The observed reduction in non-HF cardiovascular hospitalizations may reflect a global stabilization of the patient’s status. By improving the sGC-cGMP pathway and reducing HF-related instability—which often triggers other cardiovascular events like arrhythmias or type 2 myocardial infarction—vericiguat and optimized GDMT likely contribute to a broader reduction in cardiovascular risk. Meanwhile, less urgent adjustments of oral diuretics remained unchanged. The proportion of patients receiving diuretics and their mean doses were stable throughout follow-up, highlighting a gap in evidence regarding the clinical impact of decompensations managed solely with increases in oral diuretics.

In VICTORIA, vericiguat reduced the combined risk of cardiovascular death and HF hospitalizations, primarily by lowering HF hospitalizations.^12^ In contrast, the VICTOR trial reported no benefit in patients without recent worsening of HF.^34^ Real-world studies generally report lower event rates than VICTORIA, although differences in study design and baseline therapy may account for this.^12,14–16,23,32^ Among patients with recently decompensated HF, administration of vericiguat in addition to standard therapy was associated with fewer hospitalizations, potentially reflecting both optimized management of HF and the effects of vericiguat on the nitric oxide–soluble guanylate cyclase pathway.^11^

In our study, 96% of patients initiated vericiguat at 2.5 mg daily, with 64% reaching the 10-mg dose by 12 months. By comparison, 90% of patients in the VICTORIA trial reached the 10-mg dose, compared with 50–80% in other real-world studies.^12,14–16,24,32^ Vericiguat was generally acceptably tolerated, with a discontinuation rate of 13.4% at one year. Discontinuation was most commonly due to symptomatic hypotension. Higher baseline NT-proBNP was associated with an increased likelihood of discontinuation, whereas older age, higher systolic blood pressure, and higher heart rate were linked to continued treatment, suggesting that patients with more advanced HF are less likely to maintain therapy and derive limited benefit, highlighting the potential value of earlier intervention, highlighting the potential value of earlier intervention in the worsening HF trajectory. Importantly, our findings indicate that vericiguat is associated with reverse remodelling and improved clinical outcomes, even within a stable quadruple-therapy framework.

Consequently, vericiguat improves remodelling and outcomes even alongside quadruple therapy. However, clinicians should strengthen monitoring in patients with extremely high NT-proBNP due to their higher risk of drug withdrawal. Safety profiles in our study were consistent with VICTORIA and previous real-world reports,^12,14–16,19,20,23,25^ and haemoglobin levels remained stable throughout follow-up.

The observational nature of VERISEC limits definitive causal inference, as the lack of a control group makes it difficult to separate the effects of vericiguat from natural clinical stabilization or closer follow-up. While the robust improvement in biomarkers and remodelling suggests a clear benefit, these findings should be viewed as hypothesis-generating. Consequently, the magnitude of the treatment effect reflects real-world clinical practice rather than the controlled certainty of a randomized trial.

The primary strength of this study is its large, prospective patient cohort. Nevertheless, several limitations should be noted. As an observational, non-randomized study without a control group, causal relationships between vericiguat and clinical outcomes cannot be established. Specifically, while the use of quadruple therapy remained stable during follow-up, the observational nature of the study means that the specific incremental benefit of vericiguat relative to background therapy cannot be definitively isolated. Furthermore, comparisons of event rates before and after inclusion are subject to inherent selection bias and regression to the mean. Additionally, the lack of a parallel control group precluded the use of propensity score matching to further account for baseline clinical differences. Data were collected from routine clinical practice, which may include missing information and inter-centre variability in assessments. While tachycardia-induced cardiomyopathy is potentially reversible, it often involves incomplete recovery and significant residual risk, justifying the need for GDMT optimization.^35^ Specifically, the individual breakdown of RAASi types (ACEi/ARB vs. ARNI) was not captured. However, contemporary Spanish registries in similar specialized settings report ARNI use rates of 75–80%, suggesting high background therapy optimization. Furthermore, specific longitudinal data on potential confounders such as haemoglobin levels, iron deficiency correction, percutaneous mitral valve repair, heart transplant evaluation, or LVAD implantation were not captured, as the study focused on pharmacological optimization in the context of worsening HF. Additionally, the findings primarily reflect patients treated in specialized HF units in Spain and may not be generalizable to other settings. Finally, the 1-year follow-up limits assessment of longer-term effects on survival and sustained ventricular remodelling.

In conclusion, the VERISEC study provides prospective real-world evidence that, in patients with HFrEF and recent decompensation—most of whom were already receiving stable and comprehensive quadruple therapy—initiation of vericiguat was associated with favourable changes in functional status, NT-proBNP levels, and left ventricular function. Additionally, we observed a positive clinical course with a low incidence of HF events during the 1-year follow-up. Treatment was generally acceptably tolerated. However, patients with more advanced HF (characterized by higher NT-proBNP) were less likely to maintain therapy due to lower tolerance, highlighting the potential value of earlier intervention in the worsening HF trajectory. While these observational findings are encouraging, further research is needed to definitively establish the long-term impact of vericiguat within the framework of contemporary quadruple therapy.

## Supplementary Material

xvag138_Supplementary_Data

## Declarations

### Data availability

The data are available upon justified request.

### Pre-registered Clinical Trial Number

None supplied.

## Appendix

*VERISEC investigators: Francisco Camacho Jurado, Ángel Iniesta Manjavacas, Sonia Mirabet, Silvia López Fernández, Juan Luis Bonilla Palomas, Marta Cobo-Marcos, Sara Corredera García, Vanesa Escolar, Juan Górriz-Magaña, Ainara Lozano, Aleix Olivella, Esther Sánchez Corral, Mikel Taibo-Urquía, and Francisco Bermúdez-Jiménez, Ricardo Martínez-Fernández.
