# Supplementary figures and images for: One-year multicenter prospective real-world study of vericiguat effectiveness and safety in Spain
Source: ESC Heart Fail. 2026 May 13;13(3):xvag138. doi: 10.1093/eschf/xvag138 (PMC13244579; doi:10.1093/eschf/xvag138)

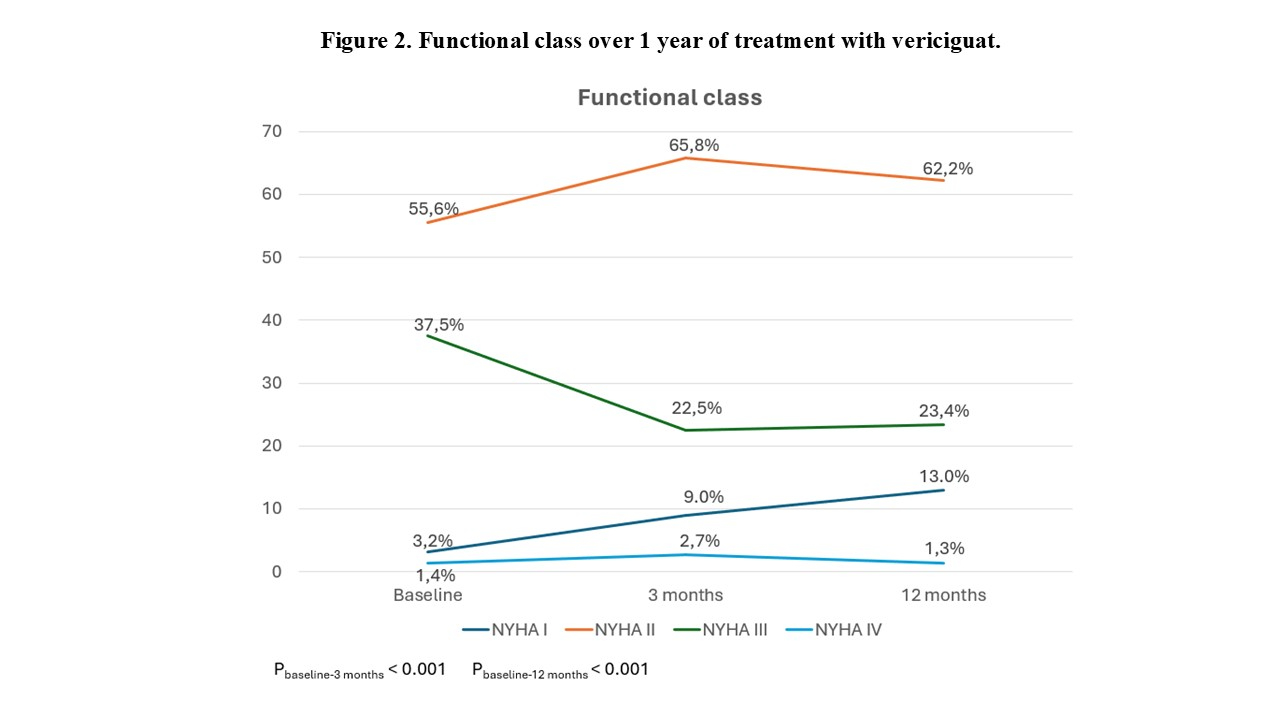

Supplement: xvag138_Supplementary_Data [file xvag138_supplementary_data.zip › Figure 1_supplementary_modified.jpg]

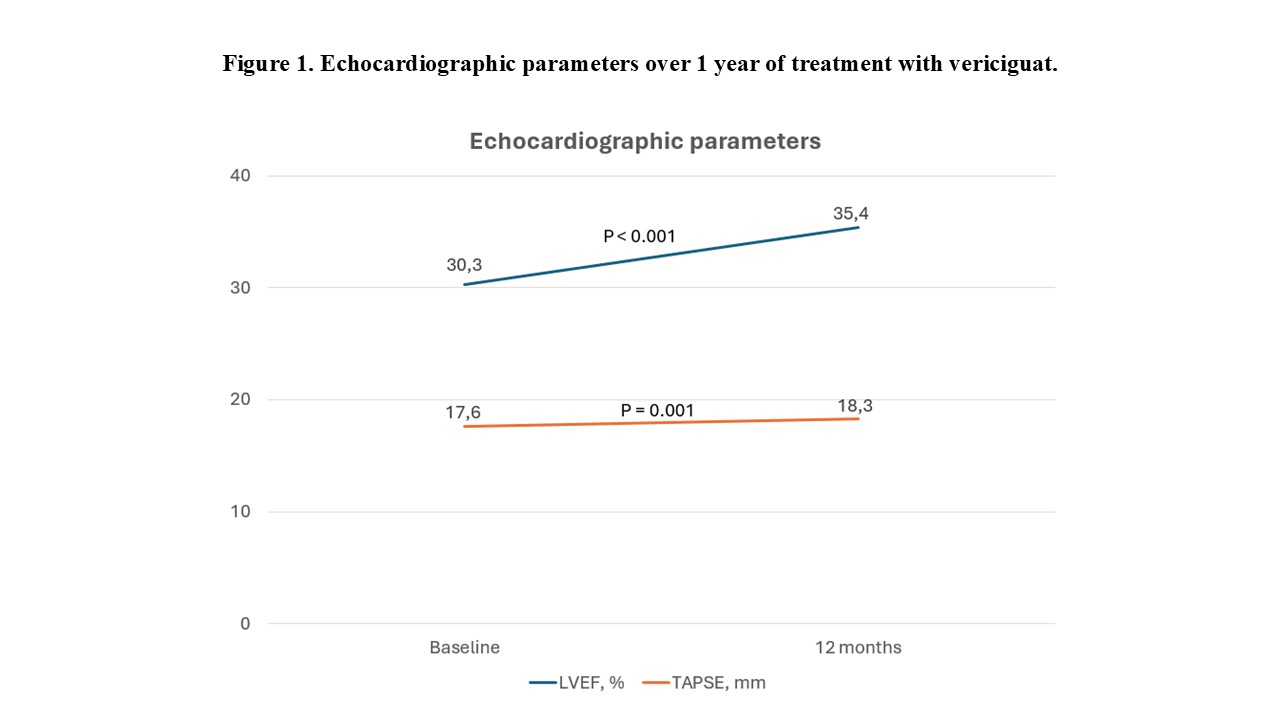

Supplement: xvag138_Supplementary_Data [file xvag138_supplementary_data.zip › Figure2_supplementary_modified.jpg]
